# Comparative analysis of endoscopic endonasal far-medial, far-lateral transcondylar and combined approaches to the foramen magnum region: volumetric analysis and quantitative insights on surgical exposure and maneuverability

**DOI:** 10.1007/s00701-026-07039-3

**Published:** 2026-09-28

**Authors:** Rachel Blue, Yan Zhou, Kamila Bond, Sonia Ajmera, Reinier Alvarez, Ross Moseley, Patrick Hosokawa, A. Samy Youssef

**Affiliations:** 1 Department of Neurosurgery, University of Pennsylvania, 3400 Spruce Street, Philadelphia, PA 19104 USA; 2 University of Colorado Anschutz School of Medicine, Aurora, CO USA; 3 Department of Neurosurgery, University of Colorado School of Medicine, Anschutz Medical Campus, Aurora, CO USA; 4 Department of Otolaryngology, University of Colorado School of Medicine, Anschutz Medical Campus, Aurora, CO USA

**Keywords:** Foramen magnum, Lower clivus, Endoscopic endonasal, Far medial, Far lateral, Transcondylar, Meningioma

## Abstract

**Background:**

Surgical access to the anterior craniovertebral junction (CVJ) and foramen magnum region remains technically challenging due to dense neurovascular anatomy and limited working corridors. Although the far-lateral transcondylar and endoscopic far-medial approaches are both employed clinically, direct quantitative comparisons of maneuverability and volumetric exposure are still lacking.

**Objective:**

To quantitatively assess and compare surgical maneuverability, anatomic limits and volume of exposure of the endoscopic far-medial, far-lateral, and combined approaches.

**Methods:**

Five latex-injected cadaveric heads underwent dissection via an endoscopic endonasal far-medial approach (EEFM), contralateral far-lateral transcondylar approach (FL), and a combined approach. Surgical freedom, horizontal and vertical angles of attack, and volumetric exposure were measured using neuronavigation and 3D CT-based segmentation. Site-specific measurements were obtained at the hypoglossal canal, jugular foramen, and vertebrobasilar junction. Statistical comparisons were made using Wilcoxon rank-sum tests.

**Results:**

The FL demonstrated significantly greater surgical freedom (363 mm^2^ vs. 6.8 mm^2^, *P* = .01), horizontal angle of attack (99° vs. 8.1°, *P* = .01), and vertical angle of attack (78° vs. 15.4°, *P* = .01) compared to the EEFM. The EEFM yielded the smallest mean total volume (5.53 ± 2.01 cm^3^), primarily concentrated in the anteromedial compartment (4.70 ± 1.67 cm^3^). The FL offered a significantly larger mean total exposure (15.12 ± 1.93 cm^3^, *p* < 0.001), dominated by posterolateral access (12.60 ± 2.16 cm^3^, *p* < 0.001). The combined approach provided the greatest overall exposure volume (18.76 ± 3.58 cm^3^), with substantial access to both the posterolateral (12.90 ± 2.32 cm^3^) and anteromedial (5.86 ± 1.95 cm^3^) compartments.

**Conclusion:**

The far-lateral approach offers superior surgical freedom and posterolateral exposure, while the far-medial endoscopic approach provides a direct but limited midline corridor predominantly anteromedial foramen magnum lesions. The combined approach maximizes volumetric access and should be considered for extensive foramen magnum lesions particularly engulfing or anterior to neurovascular structures.

## Introduction

Surgical access to the anterior craniovertebral junction (CVJ) and foramen magnum region is inherently challenging due to the anatomical complexity and proximity to critical neurovascular structures. The lower third of the clivus extends down to the foramen magnum and is bordered laterally by the occipital condyles and C1 articulation [16]. It houses key neurovascular structures including the vertebral arteries, anterior and posterior spinal arteries, lower cranial nerves (CN IX–XII) and the spinal root of the accessory nerve [8, 12, 13]. The hypoglossal nerve (CN XII) courses anterolaterally through the subarachnoid space and enters the hypoglossal canal, located between the occipital condyle and jugular foramen [22]. The vertebral artery transitions from its V3 to V4 segment near the foramen magnum, where it pierces the dura [11, 25], ascends to form the basilar artery, and gives rise to the posteroinferior cerebellar artery (PICA), with 20% arising extradurally from V3 rather than V4 [8].

The far-lateral (FL) and the endoscopic endonasal far-medial (EEFM) approaches are standard approaches to the foramen magnum region. Despite clinical use of both techniques, few studies have directly compared the quantitative exposure and maneuverability each approach provides. In particular, the relative contributions of anteromedial versus posterolateral exposure volumes and differences in surgical maneuverability, have not been rigorously analyzed in a controlled anatomical setting. In this study, we conducted a cadaver-based morphometric comparison of the FL, EEFM, and combined approaches to quantify surgical exposure volume, angles of attack, and surgical freedom at key anatomical landmarks.

## Materials and methods

Cadaveric dissections were conducted on five latex-injected human heads. Each specimen underwent an EEFM approach on one side and a contralateral FL approach, followed by a combined dissection to assess maneuverability, anatomic limits and volume of exposure. Combinations of 0°, 30°, and 45° rigid endonasal endoscopes (Karl Storz, Tuttlingen, Germany) were used during endoscopic far medial dissections and a surgical microscope was used for the transcranial far lateral approach. Dissections were performed using previously described techniques [5, 15, 22]. For analysis, thin-slice computed tomography (CT) scans were obtained prior to dissection, CT scans were registered into the Brainlab image–guided software (Brainlab AG, Munich, Germany) and points were taken after quality registration was confirmed. No IRB/patient consent was obtained for this study as it did not include any patient data, institutional approval was obtained for cadaver dissection.

## Surgical technique

### Far lateral approach

**Fig. 1:**
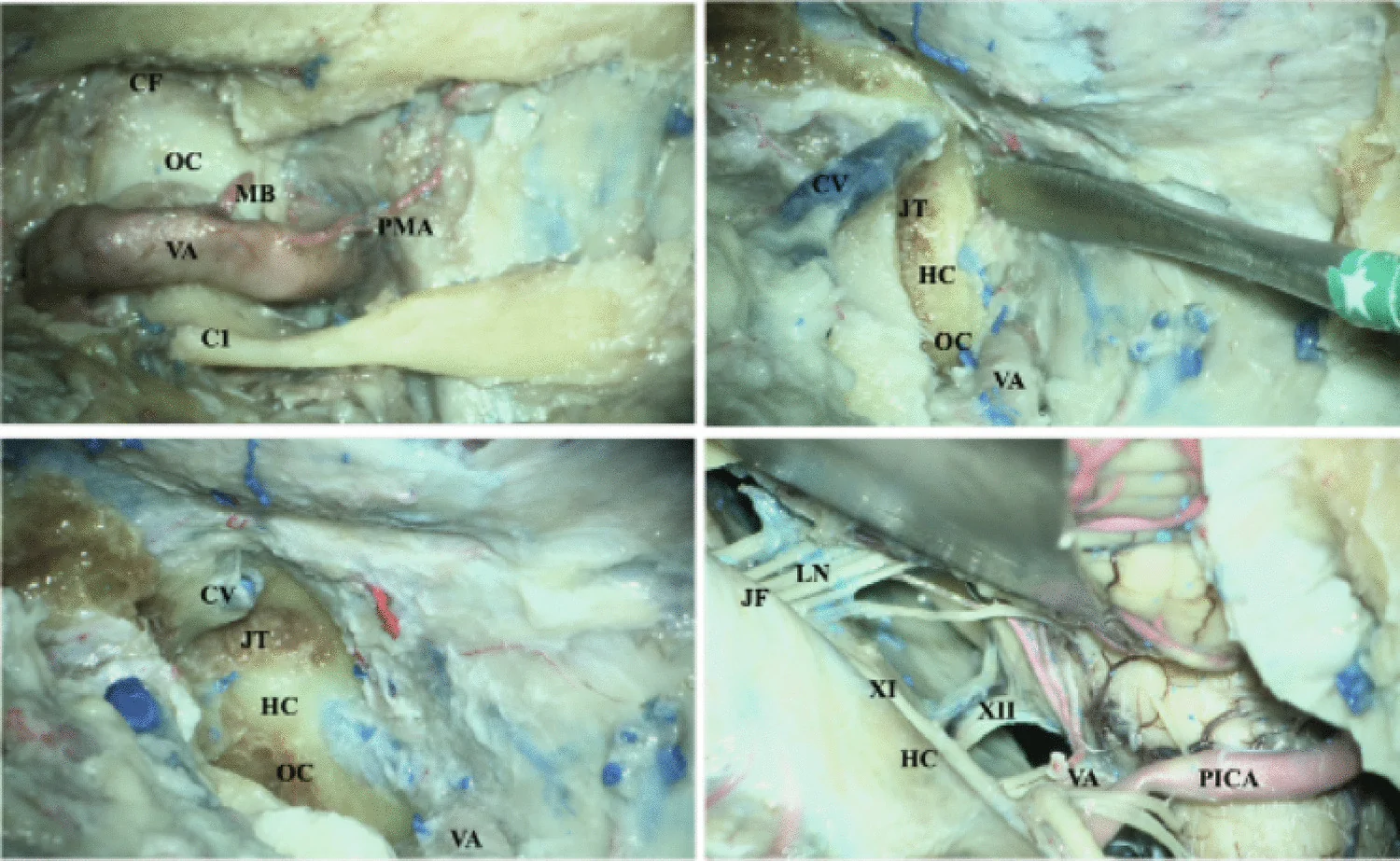
Far lateral cadaveric anatomical dissection. CF: Condylar Fossa, OC: Occipital Condyle, VA: Vertebral Artery, PMA: Posterior Meningeal Artery, CV: Condylar Vein, JT: Jugular Tubercle, HC: Hypoglossal Canal, JF: Jugular Foramen, LN: Lower Cranial Nerves, XI: Cranial Nerve 11, XII: Cranial Nerve 12

The FL approach occurs in three stages: muscular dissection, extradural dissection, and intradural dissection [24]. Our previously described interfascial dissection was utilized for the safe exposure of the vertebral artery in the suboccipital triangle [29]. Extradural dissection includes lateral suboccipital craniotomy and C1 hemilaminectomy, and removal of the lateral edge of foramen magnum. The posteromedial portion of the occipital condyle was drilled using the Primado2 NSK drill with 3-mm diamond burr (NSK Nakanishi Inc. Japan), allowing access up to the hypoglossal canal, jugular foramen, lower clivus and premedullary region [24]. The intradural stage reveals the intradural segments of vertebral artery, posterior inferior cerebellar artery, and lower cranial nerves (Fig. 1).

### Endoscopic endonasal far medial approach (EEFM)

**Fig. 2:**
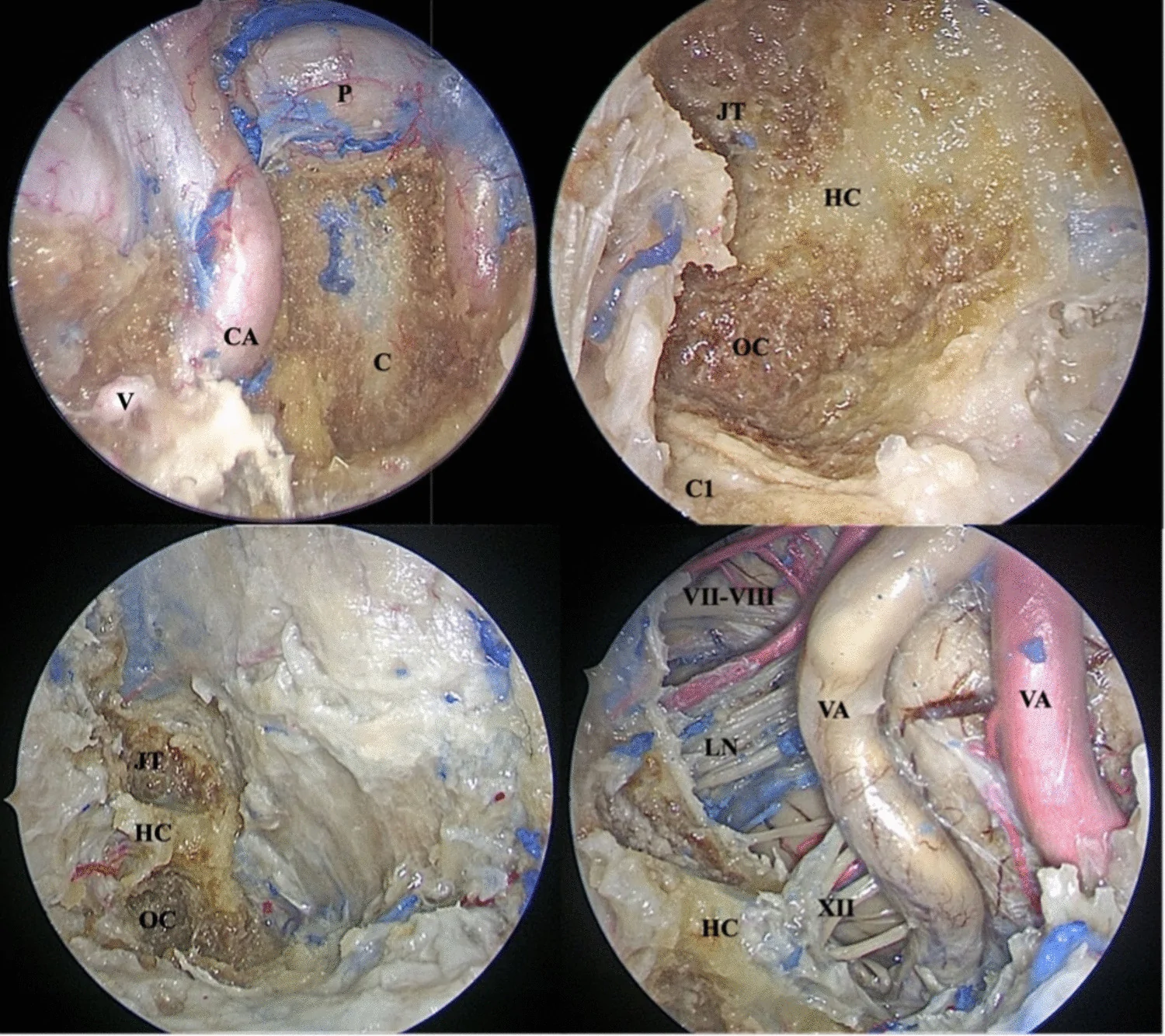
Far medial cadaveric anatomical dissection. P: Pituitary, CA: Carotid Artery, V: Vidian Nerve, C: Clivus, JT: Jugular Tubercle, HC: Hypoglossal Canal, OC: Occipital Condyle, VII-VIII: Cranial Nerves 7–8, LN: Lower Cranial Nerves, VA: Vertebral Artery, XII: Cranial Nerve 12

The EEFM is a transcondylar transjugular tubercle extension of the endoscopic endonasal transclival approach [22]. Dissection begins with a standard endoscopic endonasal approach: posterior septectomy, inferior clivectomy, and drilling up to the occipital condyle [16]. Dissection then involves removal of the atlanto-occipital joint capsule to expose the supracondylar groove [22]. Drilling the inferolateral clivus at the level of the supracondylar groove provides access to the anterior cortical wall of the hypoglossal canal, which divides the lateral clival space into the tubercular compartment superiorly and the condylar compartment inferiorly. Intradural exposure allows access to CN IX-XI as they travel towards the jugular foramen and CN XII as it passes through the hypoglossal canal (Fig. 2).

### Combined approach

Following the FL a subsequent EEFM was performed for additional analysis of exposure. As these two approaches require substantially different patient positioning, this requires staging of the approaches.

### Quantitative analysis

For each approach the length of cranial nerve exposure, degree of foramen magnum removal, surgical freedom, and angle of attack were obtained with neuro-navigation and statistically compared. Surgical freedom and angle of attack were measured both for the total surgical exposure as well as for key anatomical landmarks including hypoglossal canal, jugular foramen and vertebrobasilar junction. Total volumetric exposure was measured for each approach and compared.

#### Surgical freedom

**Fig. 3:**
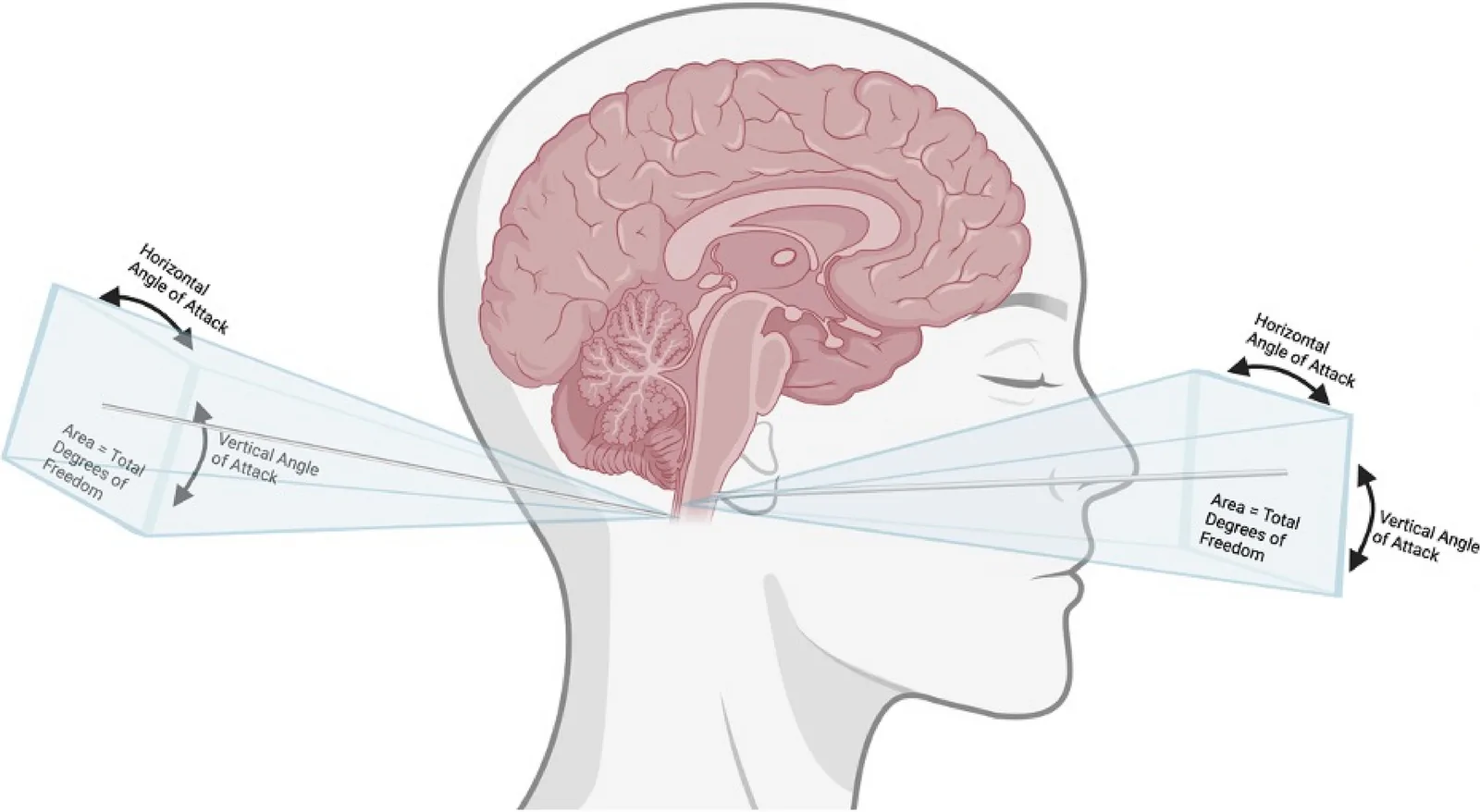
Surgical freedom and angles of attack in EEFM and FL approaches*.* The vertical and horizontal angles of attack define the limits of maneuverability and instrument reach at a fixed target point. The projected working envelope represents the total degrees of freedom, serving as a surrogate for surgical freedom. These parameters were quantitatively measured using neuronavigation for both far-medial endoscopic and far-lateral approaches to evaluate access to key anatomical targets

Surgical freedom was calculated as the maximum allowable working area at the proximal end of a 19 mm probe, with the distal end fixed at each anatomical landmark. The distal end of the probe was placed at the specific anatomical point and then moved as far medial-rostral, medial-caudal, lateral-rostral, and lateral-caudal as possible. The area was calculated by treating the quadrilateral area as 2 juxtaposed triangles. The vertical and horizontal angle of attack was calculated by the angle formed by the most medial and lateral trajectory and the most rostral and caudal trajectory, respectively (Fig. 3). Selected variables were compared across approaches with Wilcoxon Rank Sum test calculated between the two groups.

#### Volumetric assessment

**Fig. 4:**
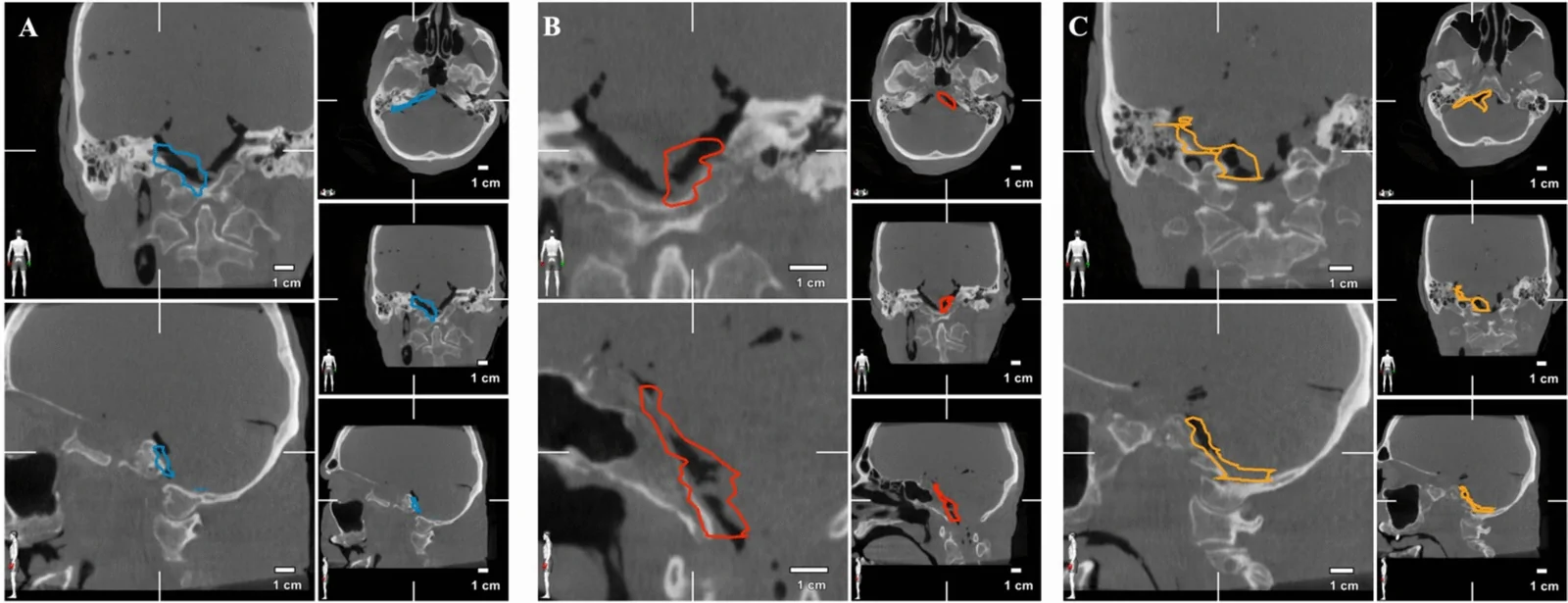
Brain lab 3D volumetric exposure between the far lateral exposure (**A**), Endoscopic endonasal far medial exposure (**B**) and combined exposure (**C**)

Volumetric exposure was assessed using a neuronavigation-guided point acquisition and manual 3D segmentation. Following each dissection, the margins of the exposed surgical corridor were identified. Discrete anatomical points outlining the perimeter of the exposure were recorded in three dimensions and imported into Brainlab Elements software, where manual segmentation was performed to define the boundaries of each surgical window. The segmented regions were reconstructed into three-dimensional volumes, which were quantified to yield total exposure volume in cubic centimeters (Fig. 4). For consistency and standardization across specimens, the sagittal midline was used as a boundary to demarcate and constrain the extent of dissection for all unilateral and combined approaches. This method allowed for accurate, reproducible comparison of surgical corridors between the FL, EEFM, and combined approaches.

In addition to calculating total volumetric exposure, each exposure was subdivided based on its anatomical relationship to the cisternal segments of the lower cranial and hypoglossal nerves. The region located anterior to the plane of the cranial nerves were defined as anteromedial**,** while region posterior to the nerves were classified as posterolateral. This allowed each exposure to be quantitatively analyzed in terms of both total volume and subregional distribution, providing additional insight into the directional reach and limitations of each approach.

## Results

### Total maneuverability

**Fig. 5:**
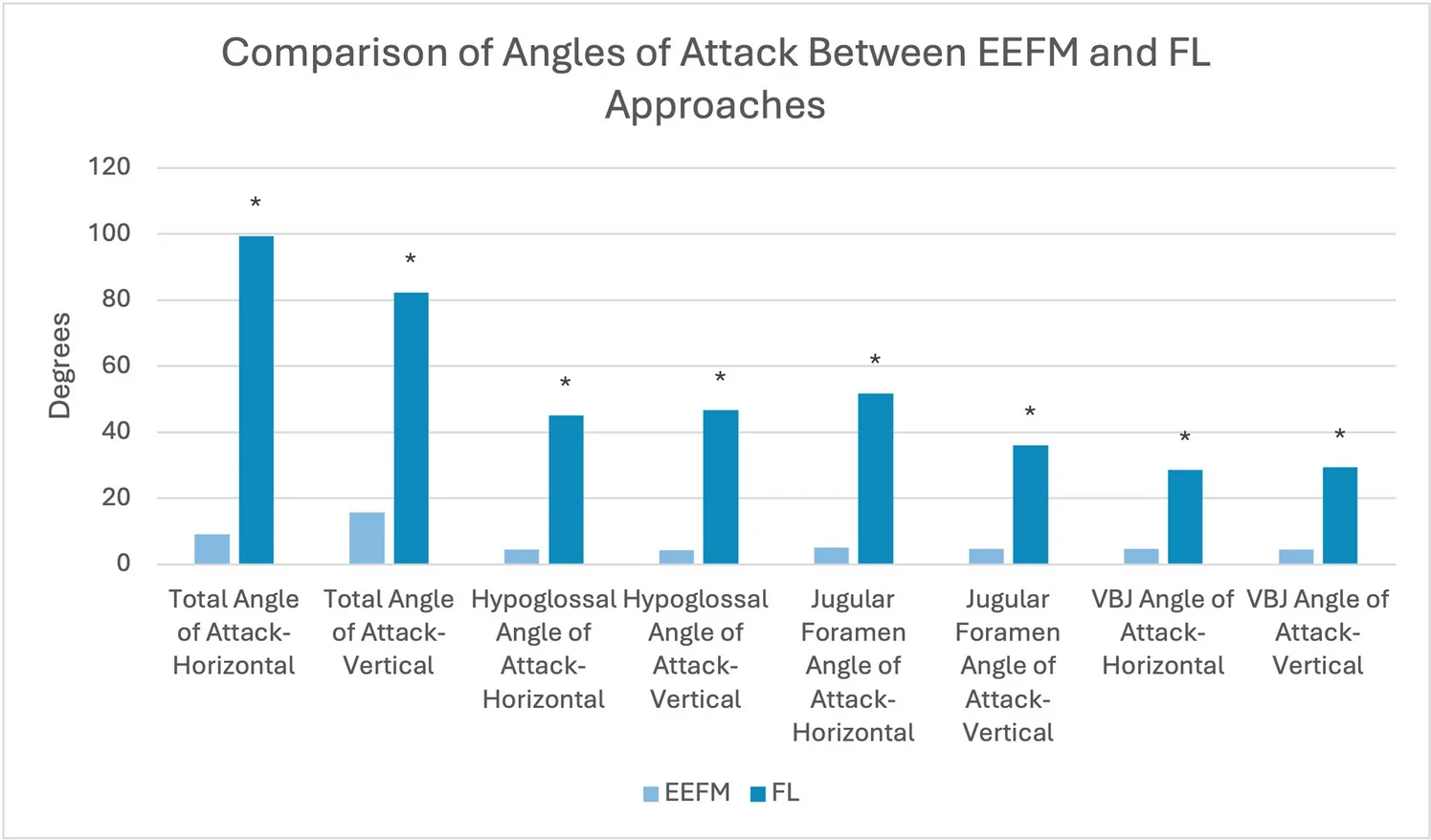
Comparison of angles of attack between far-medial endoscopic (EEFM) and far-lateral (FL) approaches. Bar graph illustrates the mean horizontal and vertical angles of attack (in degrees) at the hypoglossal canal, jugular foramen, and vertebrobasilar junction. * indicates statistical significance

Quantitative analysis demonstrated significant differences in maneuverability and angles of attack between the FL and EEFM approaches (Fig. 5). The total surgical freedom provided by the FL approach was significantly larger at 363 mm^2^ (361–368 mm^2^) compared to 6.8 mm^2^ (6.7–7.6 mm^2^) in the EEFM approach (*p* = 0.01). The FL approach provided substantially greater horizontal and vertical angles of attack compared to the EEFM approach across all anatomical landmarks. The total horizontal angle of attack was significantly higher in the FL approach at 99° (98–101°) compared to 8.1° (6.9–11.2°) in the EEFM approach (*p* = 0.01). Similarly, the total vertical angle of attack was also significantly greater in the FL approach, measuring 78° (78–89°) versus 15.4° (13.9–17.9°) in the EEFM approach (*p* = 0.01), underscoring the superior maneuverability of the FL approach in accessing dorsolateral structures.

### Key anatomical exposure

The hypoglossal canal, jugular foramen, and vertebrobasilar junction (VBJ) were analyzed to assess surgical exposure and maneuverability at these anatomical structures. For the hypoglossal canal, the FL provided significantly greater surgical freedom at 138 mm^2^ (72–145 mm^2^) compared to 1.2 mm^2^ (1.2–1.3 mm^2^) in the EEFM (*p* = 0.01). The horizontal angle of attack was also significantly greater in the FL at 41° (35–51°) versus 4.5° (3.0–5.1°) in the EEFM (*p* = 0.01), as was the vertical angle of attack at 46° (39–53°) compared to 4.2° (3.6–4.5°) (*p* = 0.01) respectively. For the jugular foramen, the FL provided a superior surgical freedom of 101 mm^2^ (93–110 mm^2^) versus 1.5 mm^2^ (1.0–1.6 mm^2^) in the EEFM (*p* = 0.01). The horizontal angle of attack was 52° (48–56°) in the FL compared to 4.8° (4.8–5.1°) in the EEFM (*p* = 0.01), while the vertical angle of attack was 41° (34–45°) in the FL versus 4.5° (3.6–5.4°) in the EEFM (*p* = 0.01) Fig. 5A.

For the VBJ, the FL provided significantly greater maneuverability, with surgical freedom measuring 46 mm^2^ (33–65 mm^2^) compared to 1.3 mm^2^ (1.0–1.5 mm^2^) in the EEFM (*p* = 0.01). The horizontal angle of attack was also greater in the FL approach at 28° (24–32°) versus 4.5° (3.9–5.1°) in the EEFM (*p* = 0.01), while the vertical angle of attack was 26° (19–42°) in the FL compared to 3.9° (3.6–5.4°) in the EEFM (*p* = 0.01) Fig. 5A.

The length of hypoglossal (CN XII) and glossopharyngeal (CN IX) nerve exposure was similar between the two approaches, with no statistically significant differences noted. The hypoglossal nerve exposure measured 1.1 cm (1.1–1.3 cm) in the EEFM approach compared to 1.0 cm (1.0–1.1 cm) in the FL (*p* = 0.83), while glossopharyngeal nerve exposure was 1.3 cm (1.3–1.4 cm) in the EEFM and 1.3 cm (1.3–1.3 cm) in the FL (*p* = 0.58). The degree of foramen magnum removal was significantly greater in the FL, measuring 129° (128–130°) compared to 45° (44–46°) in the EEFM (*p* = 0.01).

Overall, the FL consistently provided greater surgical exposure, maneuverability, and angles of attack at key anatomical landmarks of the lower clivus and CVJ. While the EEFM provides a direct ventral trajectory to the anterior foramen magnum and lower clivus, its surgical freedom and angles of attack were significantly limited in comparison.

### Volumetric/Compartmental exposure

**Fig. 6:**
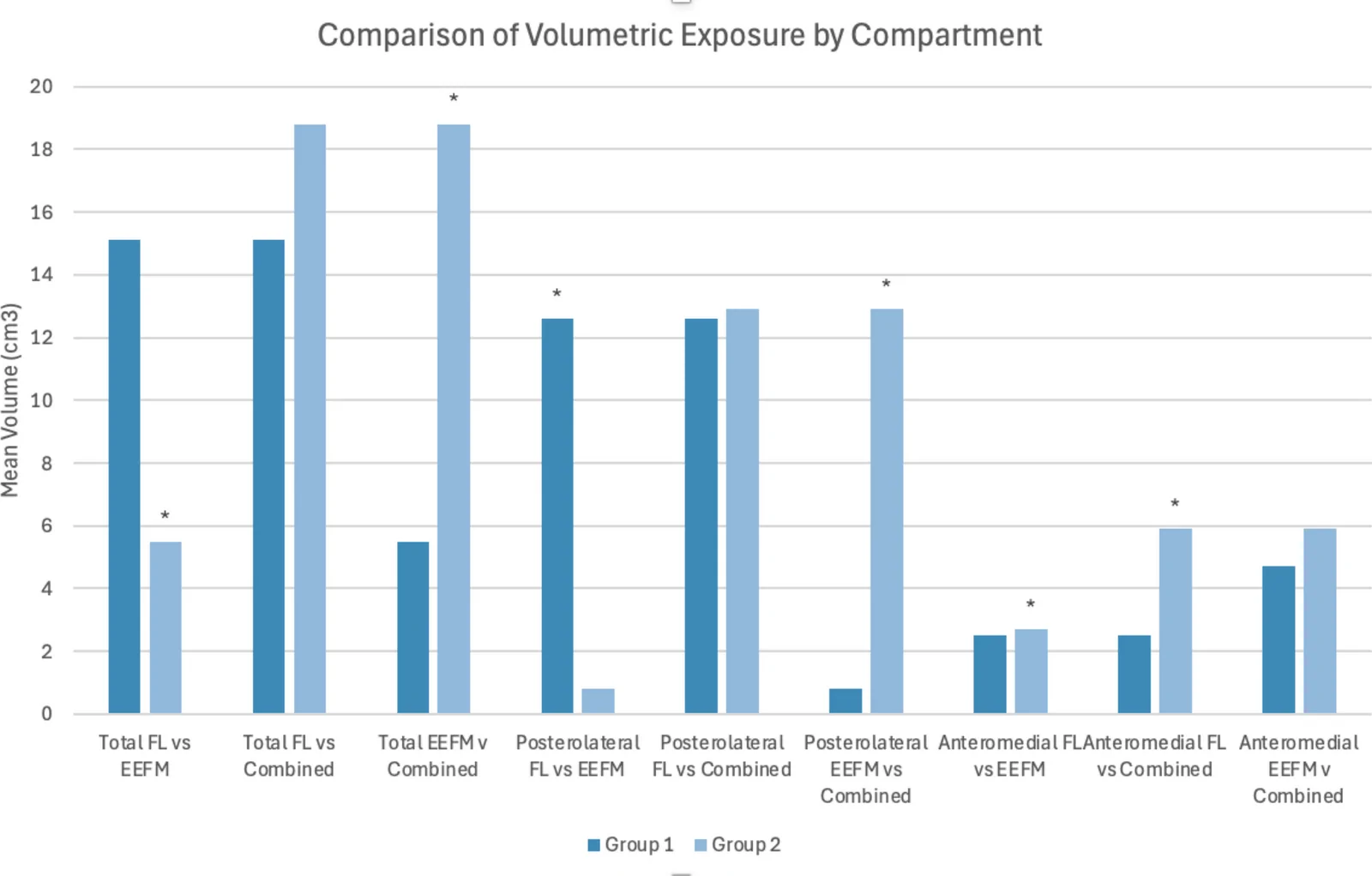
Volumetric comparison of surgical exposure between approaches to the foramen magnum region. Bars display pairwise comparisons of mean volumetric exposure (cm^3^) for total, posterolateral, and anteromedial compartments across the far-lateral (FL), endoscopic endonasal far-medial (EEFM), and combined approaches. For each paired comparison (e.g., “Total FL vs EEFM”), the darker bar denotes the first-named approach and the lighter bar the second-named approach. Asterisks (*) indicate a statistically significant difference (*p* < 0.05) between the compared approaches

Quantitative volumetric comparison among the three surgical approaches, EEFM, FL, and combined, demonstrated significant differences in total volume as well as compartment specific exposures (Fig. 6). The EEFM yielded the smallest mean total volume (5.53 ± 2.01 cm^3^), primarily concentrated in the anteromedial corridor (4.70 ± 1.67 cm^3^) with minimal posterolateral extension (0.83 ± 0.51 cm^3^). In contrast, the FL offered a significantly larger mean total exposure (15.12 ± 1.93 cm^3^, *p* < 0.001) vs. EEFM, dominated by posterolateral access (12.60 ± 2.16 cm^3^, *p* < 0.001), while anteromedial exposure was limited (2.52 ± 1.02 cm^3^, *p* = 0.038). The combined approach provided the greatest overall exposure volume (18.76 ± 3.58 cm^3^), with substantial access to both the posterolateral (12.90 ± 2.32 cm^3^) and anteromedial (5.86 ± 1.95 cm^3^) compartments. Compared to EEFM, the combined approach significantly increased total and posterolateral exposure (*p* < 0.001 for both), though the increase in anteromedial volume did not reach statistical significance (*p* = 0.37). When compared to FL, the combined approach achieved a modest but significant increase in anteromedial volume (*p* = 0.013) without a significant difference in posterolateral exposure (*p* = 0.84).

## Discussion

Our study results demonstrate that the FL approach offers significantly greater surgical freedom and horizontal and vertical angles of attack compared to the EEFM approach. Volumetrically, the FL provided significantly greater exposure than the EEFM dominated by posterolateral access while anteromedial exposure was limited. The EEFM yielded the smallest mean total volume, primarily concentrated in the anteromedial corridor with minimal posterolateral extension. The combined approach yielded the highest total exposure volume with access to both anteromedial and posterolateral compartments.

### Existing evidence on FL and EEFM

The FL approach is widely used to access the posterolateral CVJ, providing effective corridors to lesions at the lateral foramen magnum, lateral brainstem, jugular foramen, and hypoglossal canal [7, 12, 19, 21]. It has been widely employed in the resection of foramen magnum meningiomas [6–8, 12, 20] and schwannomas of lower cranial nerves and upper cervical nerves [9, 18, 21, 31, 34]. The EEFM approach evolved as an alternative, more direct approach to anterior foramen magnum lesions. Benet et al. conducted a comparative analysis with quantitative measures of brainstem exposure and surgical corridor areas and found that the EEFM has significantly larger exposure of ventromedial brainstem [5]. On the other hand, FL is more advantageous in accessing the dorsolateral brainstem with 74% exposure area and allows a larger surgical corridor from dorsolateral to ventromedial compartments through the lower cranial nerves [28]. In a more recent study, Wang et al. compared the FL to the subtonsillar and endoscope-assisted subtonsillar approaches and found FL provided the largest surgical area defined by jugular foramen, hypoglossal canal, and their most distal accessible points on the clivus with greatest angle of attack at jugular foramen, hypoglossal canal and the bifurcation point of PICA and vertebral artery [32].

Variations of the FL approach, including basic, transcondylar, and supracondylar extensions, allow the degree of condylar and jugular tubercle removal to be tailored to lesion size, location, and extension across the CVJ [24, 30]. Endoscope-assisted far-lateral techniques have also been described, using the endoscope to improve visualization around the ventral brainstem and craniovertebral junction without additional bone removal; quantitative cadaveric data suggest this can widen the surgical corridor and angle of attack achievable through a standard FL exposure [2, 33]. In experienced hands, these FL variations can extend the indications of the transcranial route to selected anterolateral and even limited anteromedial CVJ lesions, narrowing the range of pathology for which an endonasal corridor is uniquely advantageous. Invasiveness however, is multifactorial and in experienced hands, the FL approach can also be applied to selected anterolateral craniovertebral junction lesions, and endonasal corridors carry their own risks, including a comparatively higher rate of postoperative cerebrospinal fluid leak [2]. The relevant question is therefore not simply whether the EEFM is less invasive, but whether it achieves exposure and maneuverability at least equivalent to the more established FL approach.

### Current study

Quantitatively, the FL demonstrated significantly greater surgical freedom (*p* = 0.01), horizontal angle of attack (*p* = 0.01), and vertical angle of attack (*p* = 0.01) when compared to the EEFM approach. These findings confirm that the FL approach affords superior surgical freedom at all critical anatomical landmarks.

Volumetric analysis further supports these distinctions. The FL provided a significantly greater exposure volume (*p* < 0.001), with most of the exposure concentrated in the posterolateral compartment (*p* = 0.0001). In contrast, the EEFM primarily accessed the anteromedial region (*p* = 0.04), with limited posterolateral reach. This pattern underscores its advantage in direct ventral access while highlighting its limitations in lateral extension and surgical freedom.

The EEFM offers a direct route to the anterior CVJ, including lower clivus, medial jugular tubercle, and anterior foramen magnum, without an external incision or posterior fossa muscular dissection; this must be weighed, however, against the recognized risks of endonasal corridors including postoperative CSF leak [1, 22]. It has been described in treating extracranial hypoglossal schwannoma [17], chordoma [16, 26], and chondrosarcoma [4, 23, 27]. However, its limited angles of attack, narrow corridor and slow learning curve present challenges for addressing lesions with lateral extension, particularly near the occipital condyle, where the risk of cranial nerve injury increases [5, 10, 22].

The combined approach, integrating both FL and EEFM approaches, yielded the highest total exposure volume, with substantial access to both posterolateral and anteromedial compartments. Compared to the FL approach, the combined approach achieved a modest but statistically significant increase in anteromedial volume (*p* = 0.01). This suggests that while the FL approach offers extensive unilateral exposure, adding an EEFM corridor enables more circumferential dissection, which can be particularly useful in tumors that cross the midline or encase neurovascular structures. This incremental anteromedial gain, however, must be weighed against the practical costs of the combined approach: staging under separate patient positioning, increased total operative time, anesthesia exposure, and perioperative morbidity relative to either approach performed alone. For lesions with only modest anteromedial extension beyond the reach of the FL, an isolated FL approach, potentially supplemented with endoscopic assistance to enhance ventral visualization without additional bone removal, may achieve comparable exposure without the added burden of a second staged procedure [33].

These data provide quantitative support for a nuanced, anatomy-driven selection strategy when navigating the complex anatomy of the lower clivus and craniovertebral junction.

### Approach selection

Lesion-specific location and relation to critical neurovascular anatomy must guide approach selection. Ideally, a skull base approach should not transgress neurovascular structures. Anterior foramen magnum lesions often displace the brainstem and cranial nerves posteriorly, thus limiting lateral-to-medial reach of the FL [22]. For such lesions, the EEFM can be selected more favorably. Conversely, lesions with lateral extension beyond the medial jugular tubercle may exceed the working limits of the EEFM route, particularly when critical structures obscure the medial-to-lateral trajectory [5, 10, 22]. However, erosive or expansile anteromedial lesions enhance the EEFM access. FL should be selected for predominantly lateral and posterolateral lesions. Most anterior and lateral intradural lesions grow below the vertebral artery, displacing cranial nerves superiorly and the cord posteriorly, thus necessitating more medial drilling to improve exposure [8]. In our recent classification of foramen magnum meningiomas, we identified four types in relation to neurovascular structures [14]. Small tumors anterior to the neurovascular structures (Type 3) are better approached with an EEFM approach. In case of larger lesions or when the neurovascular structures are encased by tumor such as in Type 4, consideration should be given for a combined/staged EEFM and FL approaches depending on intraoperative difficulty. This anatomically driven selection algorithm is corroborated by clinical outcome data as Bassiouni et al. reported favorable long-term neurological outcomes and low recurrence following microsurgical resection of anterior and anterolateral foramen magnum meningiomas via a posterolateral suboccipital retrocondylar (far-lateral) approach, providing a clinical correlate to the exposure advantages of the FL demonstrated in our cadaveric model [3].

### Study limitations and generalizability

This is an anatomical study in specimens that lack any regional pathology, which differ from clinical and in vivo settings. However, objective anatomical data provide quantitative support for a nuanced, anatomy-driven selection strategy for a surgical approach when navigating the complex anatomy of the lower clivus and craniovertebral junction in the foramen magnum region.

## Conclusion

The FL and EEFM approaches have distinct advantages and limitations in accessing the lower clivus, hypoglossal canal, and jugular foramen in the foramen magnum region. The FL approach offers superior exposure and surgical freedom to posterolateral compartment, while the endoscopic EEFM approach affords a limited, direct route to the midline and anterolateral compartment as an alternative rather than a uniformly less invasive option, given its own set of approach related risk factors, such as CSF leak. The combined approach achieves the largest total exposure and can be considered for extensive multicompartmental lesions encasing neurovascular structures. The optimal approach selection should be tailored to each pathology particularly location, lateral extension, and displacement of neurovascular structures, to balance maximal exposure with minimal risk to such critical structures.

## Data Availability

No datasets were generated or analysed during the current study.
